# Supplementary material for: Rescue of neuropsychiatric phenotypes in a mouse model of 16p11.2 duplication syndrome by genetic correction of an epilepsy network hub
Source: Nat Commun. 2023 Feb 17;14:825. doi: 10.1038/s41467-023-36087-x (PMC9938216; doi:10.1038/s41467-023-36087-x)
Supplement: Supplementary file 1 — Supplementary Information [file 41467_2023_36087_MOESM1_ESM.pdf]

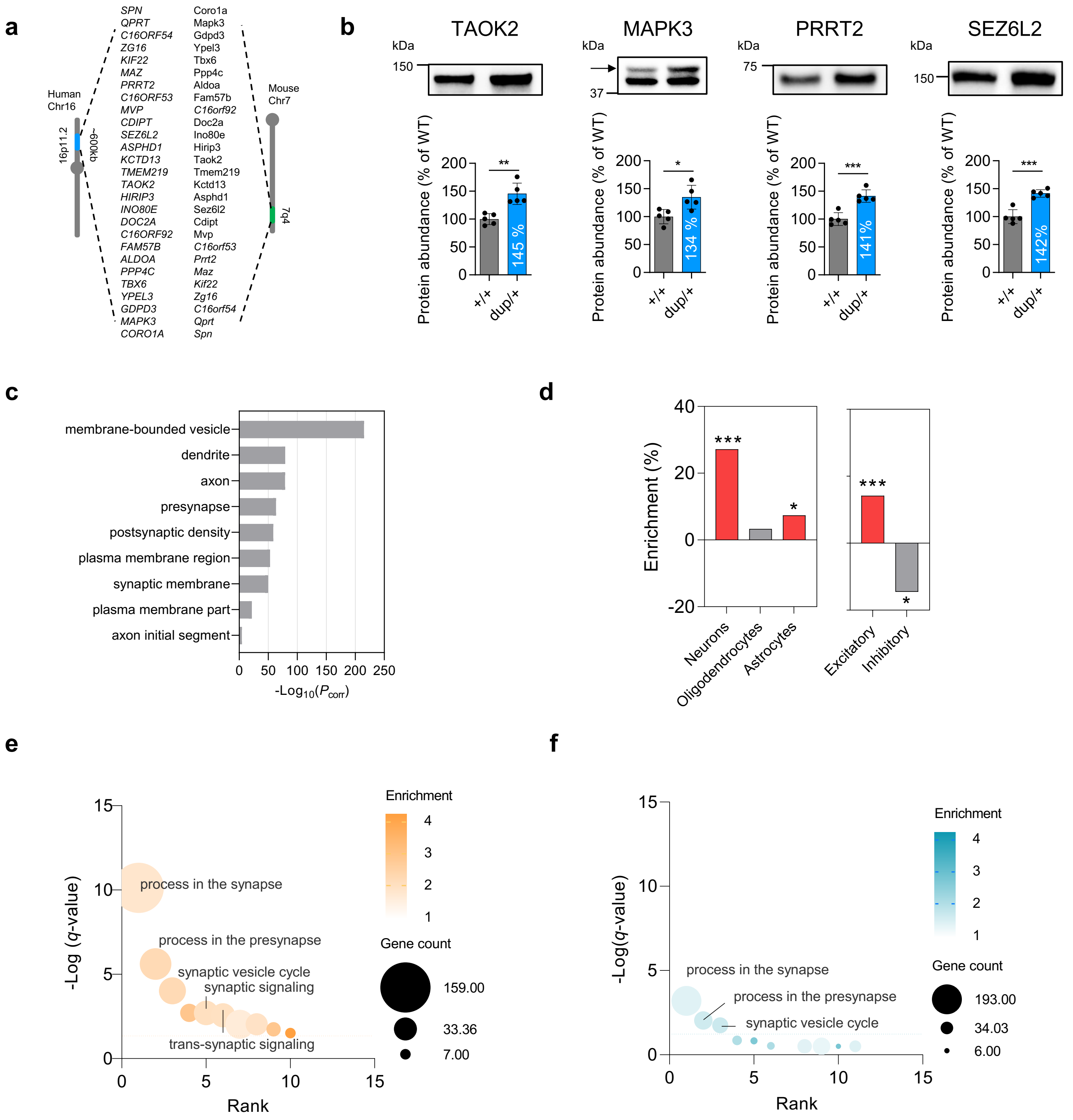
a
Human Chr16
16p11.2
~600kb
SPN
QPRT
C16ORF54
ZG16
KIF22
MAZ
PRRT2
C16ORF53
MVP
CDIPT
SEZ6L2
ASPHD1
KCTD13
TMEM219
TAOK2
HIRIP3
INO80E
DOC2A
C16ORF92
FAM57B
ALDOA
PPP4C
TBX6
YPEL3
GDPD3
MAPK3
CORO1A
Coro1a
Mapk3
Gdpd3
Ypel3
Tbx6
Ppp4c
Aldoa
Fam57b
C16orf92
Doc2a
Ino80e
Hirip3
Taok2
Tmem219
Kctd13
Asphd1
Sez6l2
Cdipt
Mvp
C16orf53
Prrt2
Maz
Kif22
Zg16
C16orf54
Qprt
Spn
Mouse Chr7
7q4
b
TAOK2
MAPK3
PRRT2
SEZ6L2
kDa
150
37
75
Protein abundance (% of WT)
+/+
dup/+
145 %
134 %
141%
142%
c
membrane-bounded vesicle
dendrite
axon
presynapse
postsynaptic density
plasma membrane region
synaptic membrane
plasma membrane part
axon initial segment
-Log10(Pcorr)
d
Enrichment (%)
Neurons
Oligodendrocytes
Astrocytes
Excitatory
Inhibitory
e
-Log (q-value)
process in the synapse
process in the presynapse
synaptic vesicle cycle
synaptic signaling
trans-synaptic signaling
Rank
Enrichment
Gene count
159.00
33.36
7.00
f
-Log(q-value)
process in the synapse
process in the presynapse
synaptic vesicle cycle
Rank
Enrichment
Gene count
193.00
34.03
6.00

Supplementary Fig. 1. Biochemical validation of *16p11.2*$^{dup/+}$ mouse model and gene set analysis of membrane proteome. (a) Illustration of the 27 protein coding genes in the human 16p11.2 chromosomal region and the conserved syntenic region on mouse chromosome 7q4. (b) Western blot showing increased expression of four proteins from the duplicated 16p11.2 region (n=5 mice) (TAOK2, p = 0.00155; ERK1, p = 0.0142; PRRT2, p = 0.00042; SEZ6L2, p = 0.000176). Closest molecular weight marker is shown, in kDa. Arrow indicates location of ERK1 band (bottom is ERK2). Data are shown as mean ± s.e.m., two-tailed t-test. (c) Gene ontology (GO) analysis showing enriched cellular compartment terms of proteins detected in the full P2 membrane fractions. P_{corr} = Benjamini-Hochberg corrected p value. (d) Gene set analysis of showing overrepresentation of proteins that are enriched in excitatory neurons (Neurons, $p = 1.46 \times 10^{-10}$, Astrocytes, p = 0.049; Excitatory, p = 0.00096; Inhibitory, p = 0.017). Data are presented as percentage enrichment and p-values from a two-sided hypergeometric test (e) SynGO analysis showing upregulated proteins are enriched for synaptic pathways, with bias for presynaptic functions (one-sided Fisher's Exact test with FDR-correction). (f) Downregulated proteins are much less enriched for synaptic ontologies compared to upregulated proteins (one-sided Fisher's Exact test with FDR-correction). *p < 0.05, **p< 0.01, ***p< 0.001.

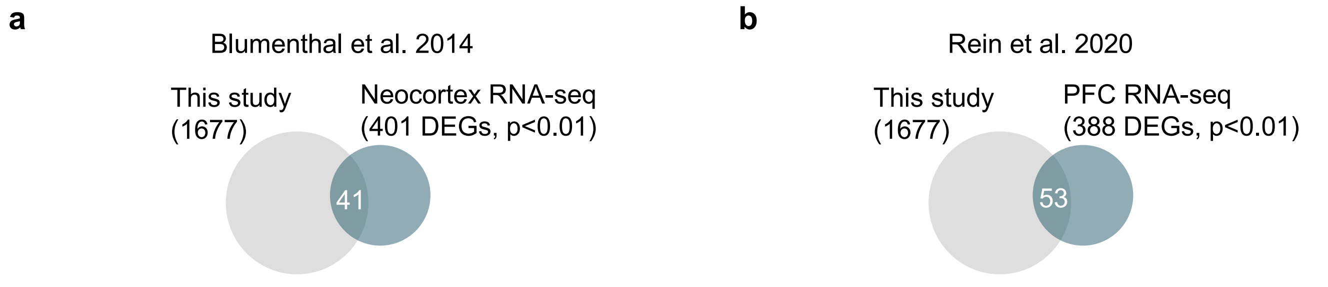

c

RNA-seq study	PMID	Gene sets	Total DEGS (p<0.01)	DEGs in membrane proteome	Overlap with 16p11.2dup/+ membrane proteome (this study)		
					All (1677)	Up (659)	Down (1024)
Blumenthal et al. 2014	24906019	All	401	98	41	20	21
		Up	181	42	14	9	5
		Down	220	56	27	11	16
Rein et al. 2021	32099100	All	388	104	53	29	24
		Up	111	11	3	2	1
		Down	277	93	50	27	23

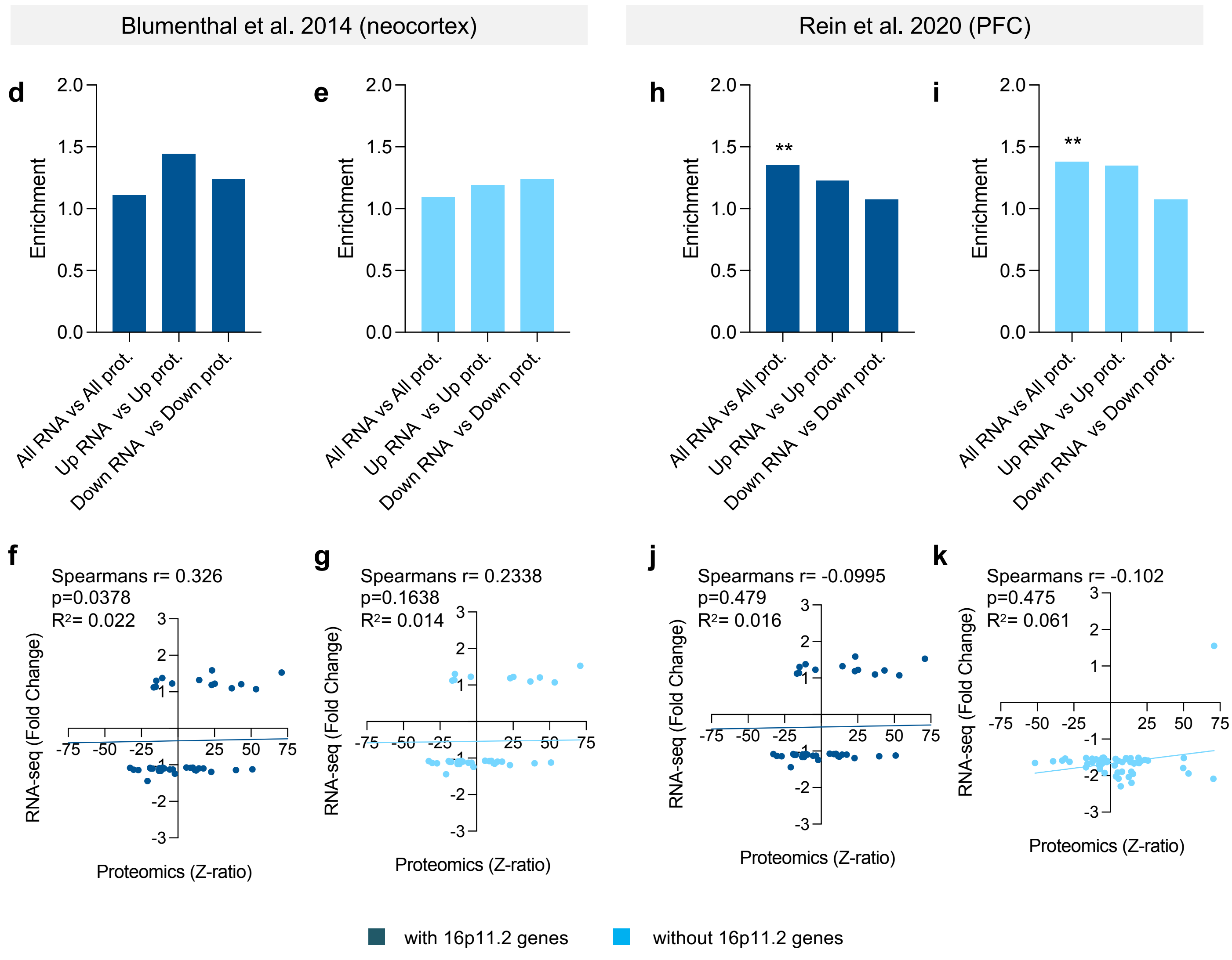

Supplementary Fig. 2. Correlating membrane proteomics with RNA-seq in the 16p11.2dup/+ mouse model. (a) Venn diagram showing overlap between membrane proteome dataset and RNA-seq from whole neocortex[39] (b) Venn diagram showing overlap between membrane proteome dataset and RNA-seq from prefrontal cortex (PFC)[30]. (c) Table showing number genes in different gene sets created from RNA-seq datasets and their corresponding overlap in the membrane proteome dataset. (d-i) Gene set enrichment analysis (GSEA) comparing proteomic and transcriptomic datasets in 16p11.2$^{dup/+}$ mice (results from a two-sided hypergeometric test) (d) GSEA in neocortex RNA-seq dataset with 16p11.2 genes included. (e) GSEA in neocortex RNA-seq dataset with 16p11.2 genes excluded (f) Correlation analysis with neocortex RNA-seq dataset including 16p11.2 genes. (g) Correlation analysis with neocortex RNA-seq dataset with 16p11.2 genes removed. (h) GSEA in PFC RNA-seq dataset with 16p11.2 genes included (All RNA vs. All protein, p = 0.0037). (i) GSEA in PFC RNA-seq dataset with 16p11.2 genes excluded (All RNA vs. All protein, p = 0.0024). (j) Correlation analysis with PFC RNA-seq dataset including 16p11.2 genes. (k) Correlation analysis with PFC RNA-seq dataset without 16p11.2 genes. R and P-values are from a nonparametric spearman's correlation. R^2 values indicate goodness of fit from a linear regression analysis. Abbreviations: GSEA; gene set enrichment analysis.

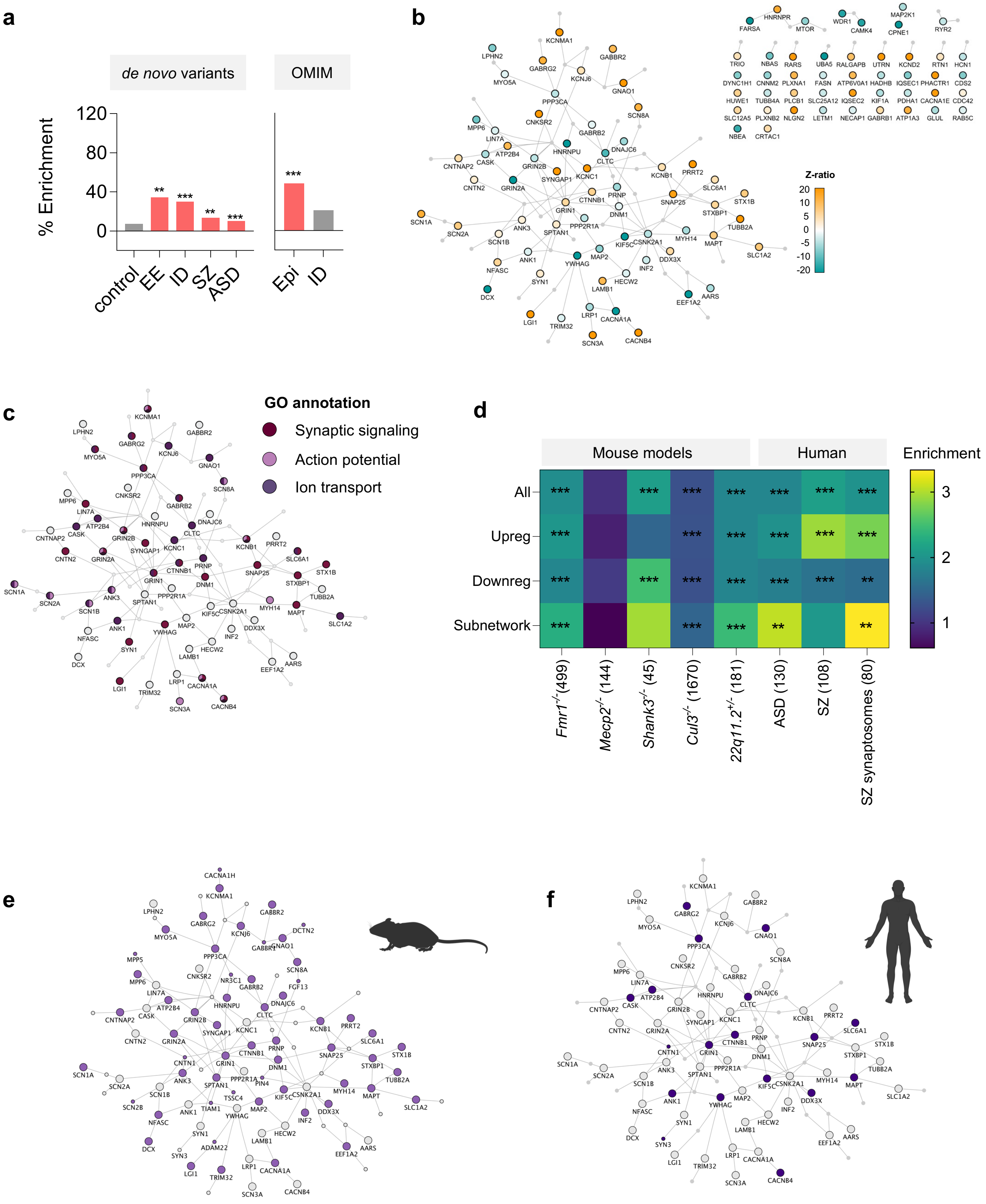
a
de novo variants
OMIM
% Enrichment
120
80
40
0
**
***
**
***
***
control
EE
ID
SZ
ASD
Epi
ID
b
Z-ratio
20
10
5
0
-5
-10
-20
c
GO annotation
Synaptic signaling
Action potential
Ion transport
d
Mouse models
Human
Enrichment
All
Upreg
Downreg
Subnetwork
Fmr1-/- (499)
Mecp2-/- (144)
Shank3-/- (45)
Cul3-/- (1670)
22q11.2+/- (181)
ASD (130)
SZ (108)
SZ synaptosomes (80)
3
2
1
e
f

Supplementary Fig. 3. An epilepsy subnetwork is dysregulated in the 16p11.2dup/+ mouse model and human neuropsychiatric disorders. (a) Gene set analysis of dysregulated proteins from 16p11.2dup/+ membrane proteome with NPD gene sets. Epilepsy-associated genes from de novo exome sequencing studies (dnSNVs) and OMIM datasets are highly enriched within the proteomic data (control, p = 0.1089; EE, p = 0.0011; ID, $p = 5.05 \times 10^{-5}$; SZ, p = 0.0027; ASD, $p = 1.83 \times 10^{-5}$; Epi (OMIM), p = 0.00024; ID (OMIM), p = 0.076.. Data are presented as percentage enrichment and p-values from a two-sided hypergeometric test. (b) The epilepsy protein:protein interaction subnetwork (n=100 nodes, from Fig. 1) represented alongside all the unconnected nodes (n=60 nodes). Colored nodes represent all the epilepsy-associated proteins that were dysregulated in the $16p11.2^{dup/+}$ membrane proteome (n=110), which were used to seed the network. Nodes are color coded by z-ratio: upregulation = z-ratio>1, downregulation =z-ratio<1. Grey nodes represent retrieved data from the GeneMANIA protein interaction database. (c) Core epilepsy subnetwork with input nodes annotated for gene ontologies. (d) Heatmap of similarity between proteomic dysregulation in the $16p11.2^{dup/+}$ mouse model, NPD mouse models and human post-mortem samples. Enrichment values in the subnetwork are generally higher than in the whole membrane proteome, indicating a potential role for this network in NPD pathophysiology. All exact enrichment scores and p-values are presented in the Source Data file. (e) Illustration of epilepsy subnetwork with proteins disrupted in NPD mouse models highlighted in purple. Mouse schematic created with BioRender.com. (f) Illustration of epilepsy subnetwork with proteins disrupted in ASD and SZ are highlighted in purple. Human schematic created with BioRender.com. As in (b) large nodes represent proteins disrupted in the $16p11.2^{dup/+}$ mouse model whilst small nodes are retrieved interacting proteins from the PPI database.

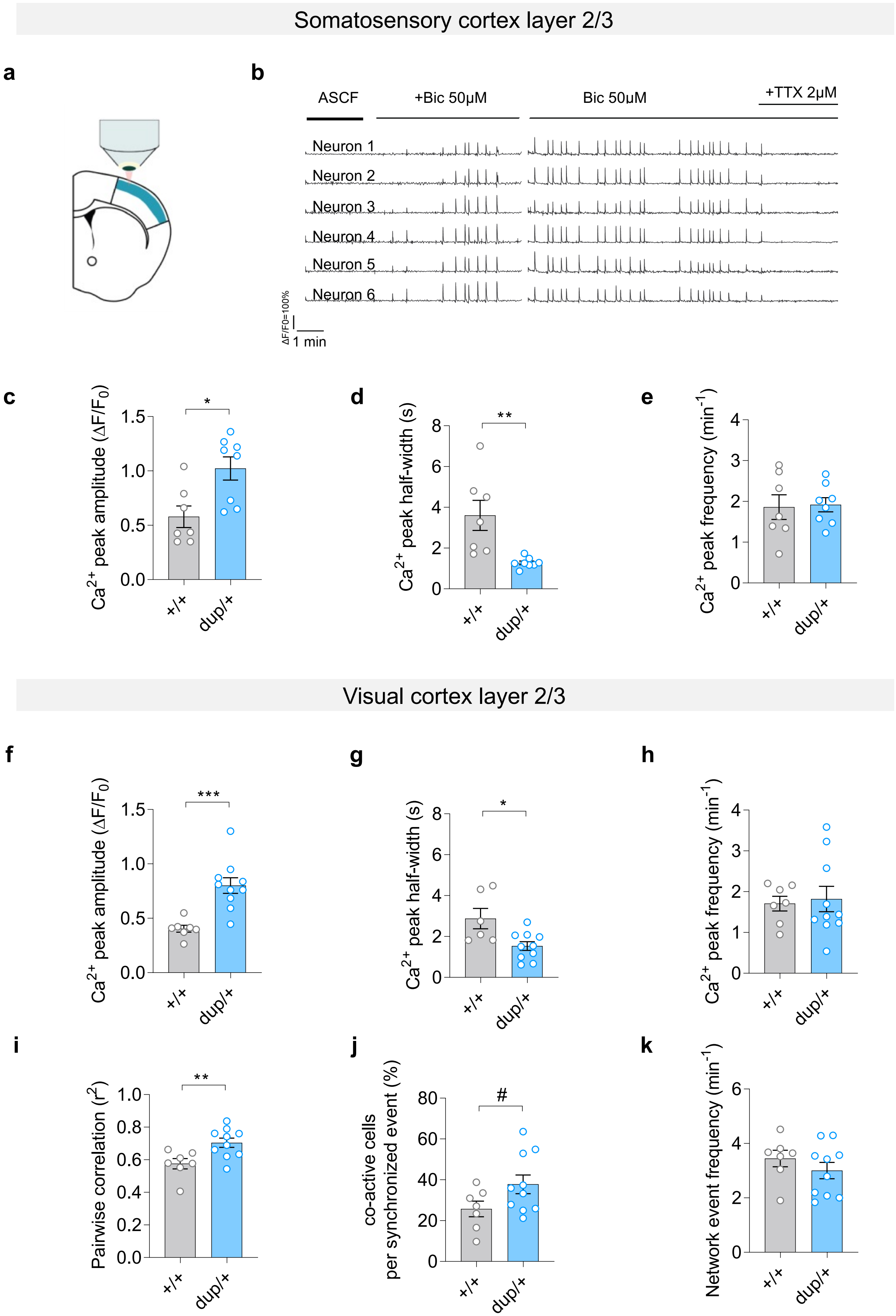
Somatosensory cortex layer 2/3
a
b
ASCF
+Bic 50µM
Bic 50µM
+TTX 2µM
Neuron 1
Neuron 2
Neuron 3
Neuron 4
Neuron 5
Neuron 6
ΔF/F0=100%
1 min
c
Ca2+ peak amplitude (ΔF/F0)
*
1.5
1.0
0.5
0.0
+/+
dup/+
d
Ca2+ peak half-width (s)
**
8
6
4
2
0
+/+
dup/+
e
Ca2+ peak frequency (min-1)
4
3
2
1
0
+/+
dup/+
Visual cortex layer 2/3
f
Ca2+ peak amplitude (ΔF/F0)
***
1.5
1.0
0.5
0.0
+/+
dup/+
g
Ca2+ peak half-width (s)
*
8
6
4
2
0
+/+
dup/+
h
Ca2+ peak frequency (min-1)
4
3
2
1
0
+/+
dup/+
i
Pairwise correlation (r2)
**
1.0
0.8
0.6
0.4
0.2
0.0
+/+
dup/+
j
co-active cells
per synchronized event (%)
#
80
60
40
20
0
+/+
dup/+
k
Network event frequency (min-1)
6
4
2
0
+/+
dup/+

Supplementary Fig. 4. Hypersynchrony and calcium dynamics in cortical circuits of the *16p11.2$^{dup/+}$* mouse model. Single neurons properties are presented in c-h, whilst multicellular network properties are presented in i-k. (a) Illustration of an acute brain slice showing the location of the primary somatosensory cortex used for two-photon calcium imaging (b) Representative traces of calcium events from individual neurons in layer 2/3 of the somatosensory cortex. Blockade of the $GABA_A$ receptor (50µM bicuculline) induced calcium oscillations that were abolished upon treatment with Na_v channel blocker tetrodotoxin (TTX, 2µM). (c-e) Calcium imaging in S1 (16p11.2$^{+/+}$, *n*=7 slices from 4 mice; 16p11.2$^{dup/+}$, n= 8 slices from 4 mice). (c) Increased calcium peak amplitude in *16p11.2$^{dup/+}$* neurons compared to WT neurons (p = 0.01) (d) Reduced calcium peak half-width (p = 0.0052) (e) No change in calcium peak frequency. (f-k) Calcium imaging in V1 (16p11.2$^{+/+}$, *n*=7 slices from 4 mice; 16p11.2$^{dup/+}$, n= 10 slices from 5 mice) (f) Increased calcium peak amplitude (p = 0.0005) and (g) Reduced calcium peak half-width (p = 0.0125) (h) No change in calcium peak frequency in *16p11.2$^{dup/+}$* neurons of visual cortex compared to *16p11.2$^{+/+}$* brain slices. (i) Increased pairwise correlation of neurons (p = 0.0095) (j) No change in co-activation of neurons during synchronized events and (k) No change in frequency of network events in *16p11.2$^{dup/+}$* visual cortex slices compared to *16p11.2$^{+/+}$* slices (p = 0.0768). Data are shown as mean ± s.e.m. #, p = 0.0768, *p < 0.05, **p< 0.01, ***p< 0.001, two-tailed t-test.

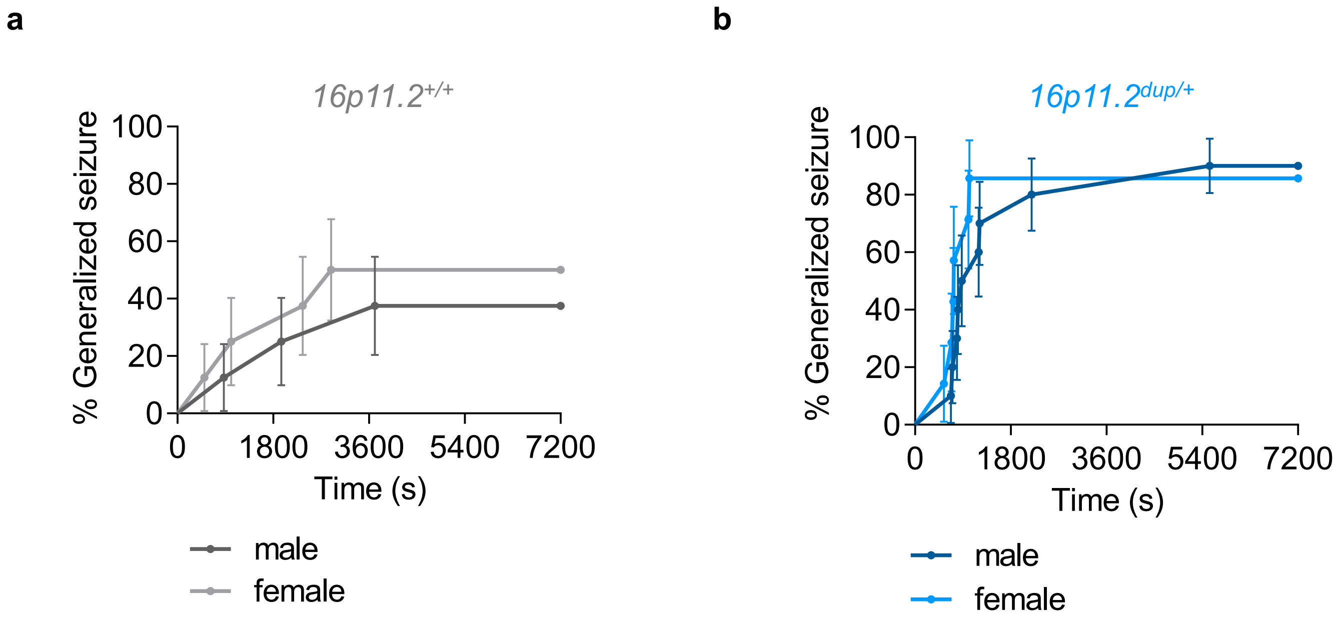

a
16p11.2+/+
% Generalized seizure
100
80
60
40
20
0
0
1800
3600
5400
7200
Time (s)
male
female
b
16p11.2dup/+
% Generalized seizure
100
80
60
40
20
0
0
1800
3600
5400
7200
Time (s)
male
female

Supplementary Fig. 5. Seizure susceptibility in male and female *16p11.2*$^{dup/+}$ mice. Kaplan-Meier curve displaying percentage of generalized tonic-clonic seizures in male and female after injection of 28mg/kg kainic acid (+/+ female n=8, +/+ male n=8, dup/+ female n=7, dup/+ male n=10. No significant differences observed in (a) *16p11.2*$^{+/+}$ mice (b) *16p11.2*$^{dup/+}$ mice when comparing males and females. Error bars represent standard error.

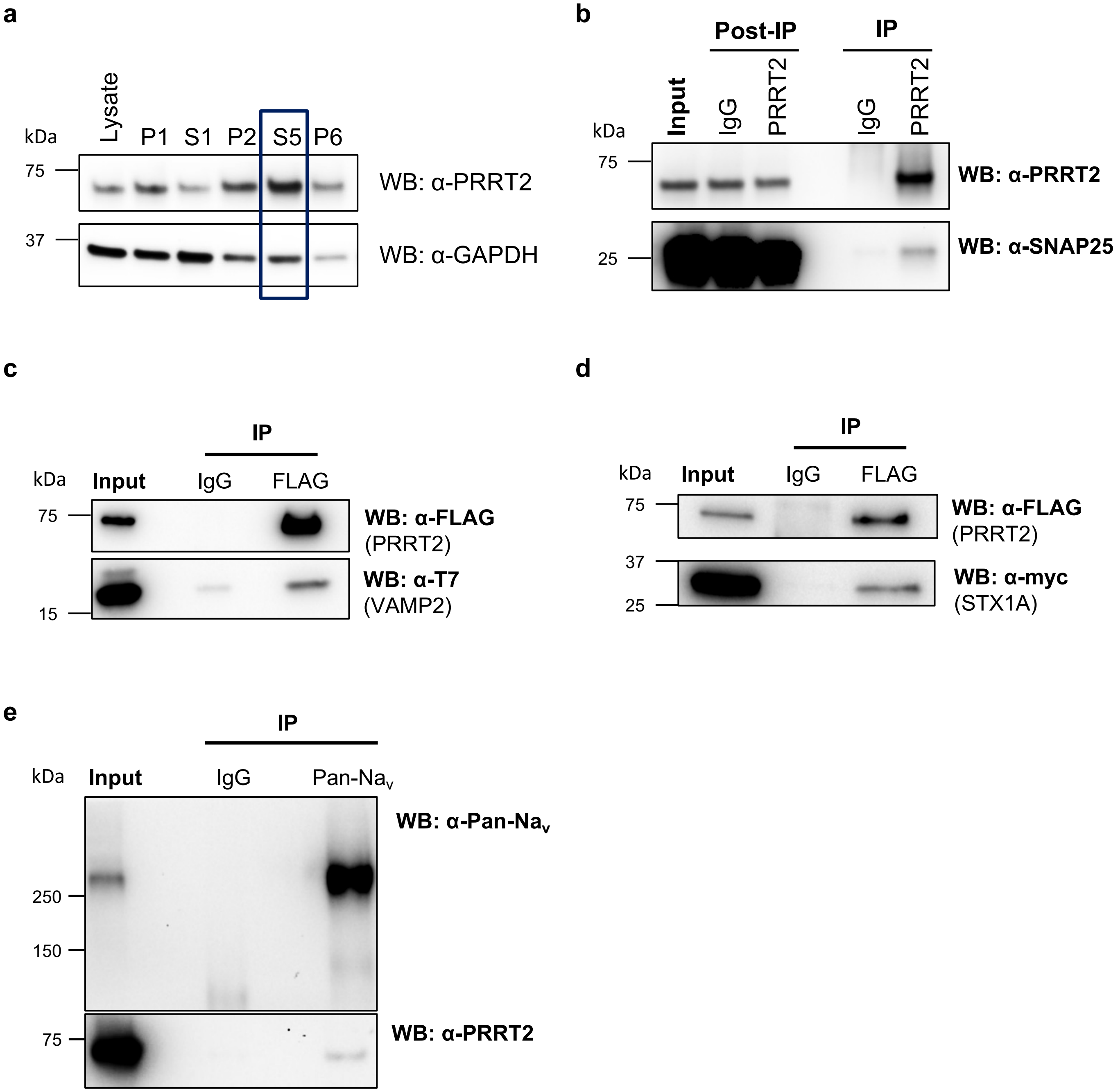
a
kDa
Lysate
P1
S1
P2
S5
P6
75
37
WB: α-PRRT2
WB: α-GAPDH
b
Post-IP
IP
Input
IgG
PRRT2
IgG
PRRT2
kDa
75
25
WB: α-PRRT2
WB: α-SNAP25
c
IP
kDa
Input
IgG
FLAG
75
15
WB: α-FLAG
(PRRT2)
WB: α-T7
(VAMP2)
d
IP
kDa
Input
IgG
FLAG
75
37
25
WB: α-FLAG
(PRRT2)
WB: α-myc
(STX1A)
e
IP
kDa
Input
IgG
Pan-Nav
WB: α-Pan-Nav
250
150
75
WB: α-PRRT2

Supplementary Fig. 6. Immunoaffinity purification of PRRT2 in mouse neocortex and validation of protein interactions. (a) Fractionation of mouse neocortex to yield washed membrane fraction (S5) enriched for PRRT2. S5 fraction was solubilized and used from downstream IAP-MS (see Methods). (b) Quality control of PRRT2 IAP and co-immunoprecipitation of SNAP25 was used as a positive control as it is a known interacting protein. PRRT2 and SNAP25 are present in the input fraction and both co-immunoprecipitate on the beads cross-linked with PRRT2 antibody but not on beads cross-linked with non-specific IgG. Both proteins remain in post-IAP supernatant (Post-IP) demonstrating that an excess of protein was used for the IAP experiment. (C-F) Validation of protein:protein interactions with PRRT2 and SNARE components using overexpression in HEK-293T cells. FLAG-PRRT2 was co-transfected and immunoprecipitated with either (c) T7-VAMP2 or (d) myc-STX1A. Both proteins co-precipitate with PRRT2. (E-F) Immunoprecipitation of ion channels in mouse neocortex (e) sodium channels (Pan-Na_V) co-immunoprecipitate with PRRT2 but not IgG. PRRT2 IPs (a-b) were performed 3 times with similar results, were as interaction validation experiments were performed once (c-e).

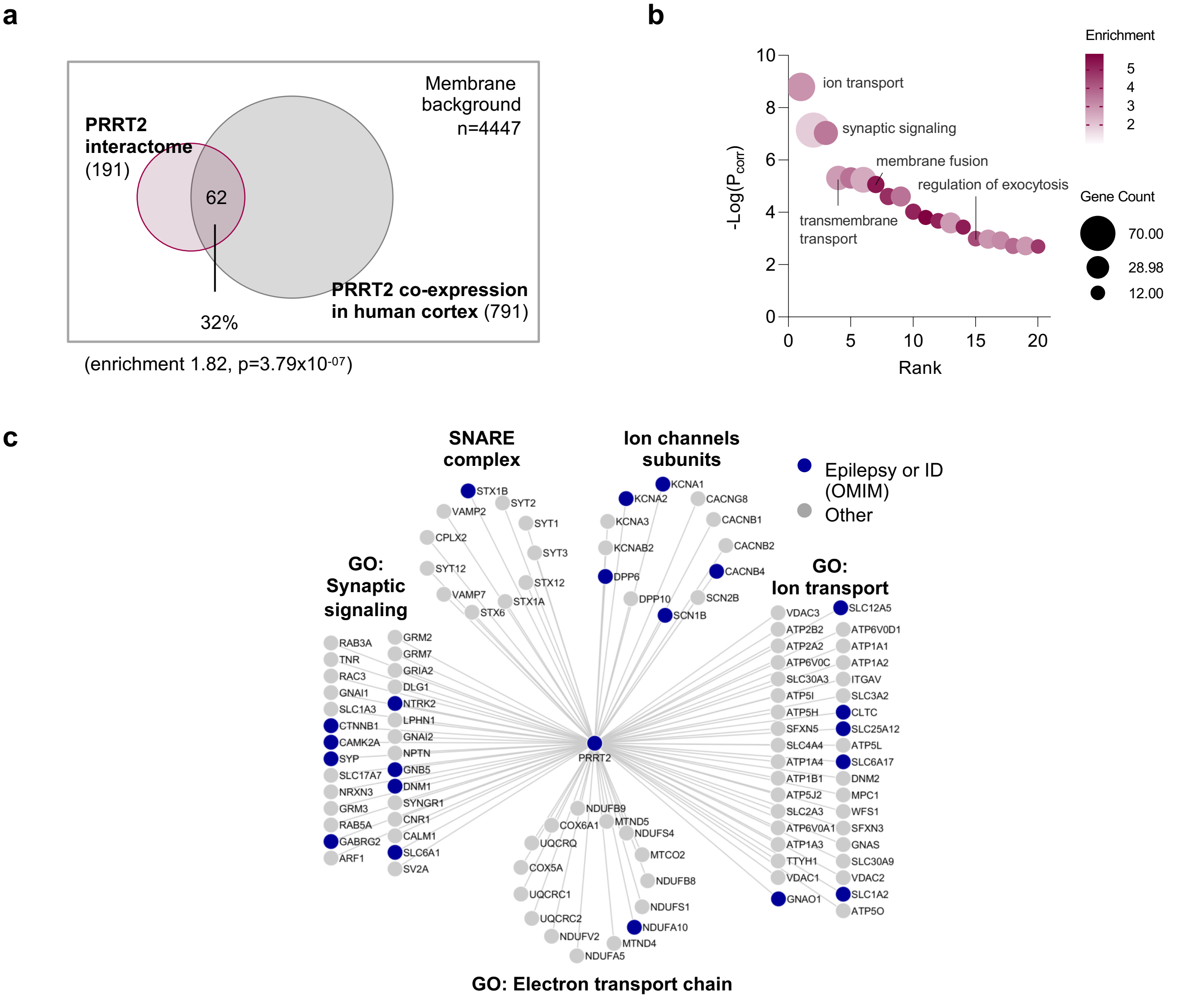

d

Patient	Allele ID	Genomic position (hg19)	Variant type	Length (bp)	Clincal features	Significance
1	247819	chr16: 29822338-29826328	copy number gain	3991	Seizures	Pathogenic
2	248234	chr16: 29822122-29826328	copy number gain	4207	Seizures	Uncertain
3	465541	chr16: 29824356-29825979	Duplication	1,624	PKD	Uncertain
4	401080	chr16: 29824311-29827202	Duplication	2,892	PKD	Uncertain

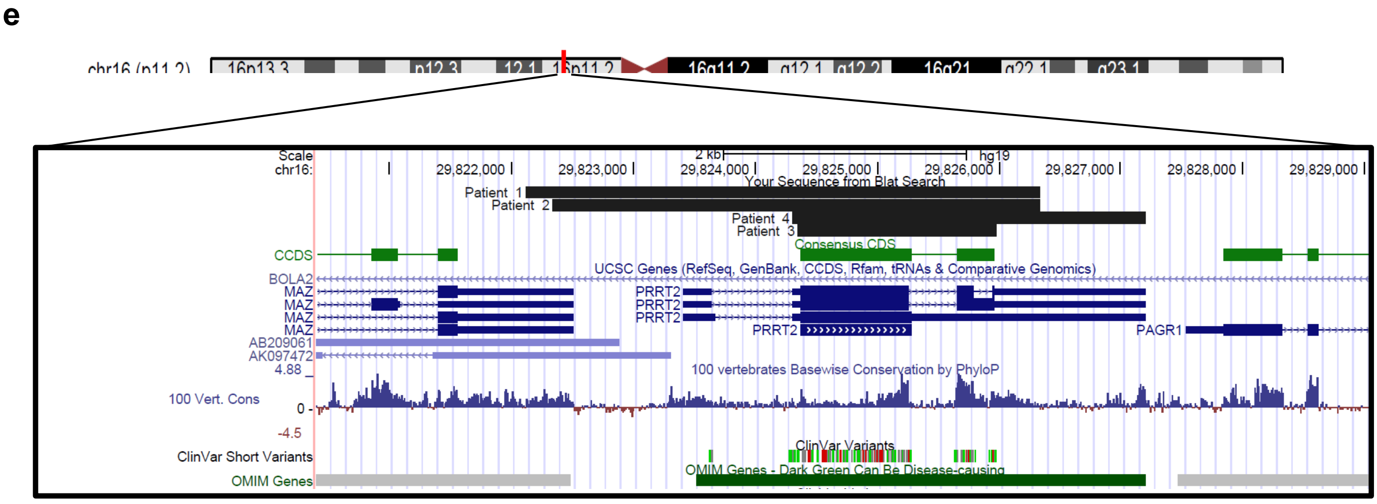

Supplementary Fig. 7. Bioinformatics of PRRT2 interactome in mouse neocortex and information on human *PRRT2* duplications from ClinVar database. (a) Overlap of PRRT2 interactome dataset and PRRT2 co-expression dataset for human neocortex. 32% of the interactome represents proteins encoded by co-expressed genes in human neocortex. The enrichment and *p*-value compared to a chance finding is indicated (results from a two-sided hypergeometric test). (b) GO analysis on PRRT2 interactome shows enrichment for ion channel and synaptic proteins (one-sided Fisher's Exact test with Benjamini-Hochberg correction). (c) Proteins from major biological functions contained in the PRRT2 interactome are presented. Categories represent a combination of manual curation from the literature and classifications based on enriched GO ontologies. Color-coded nodes are associated with ID/DD or epilepsy in OMIM. Abbreviations: dnSNV, de novo singe nucleotide variant; EE, epileptic encephalopathy; ID, intellectual disability; SZ, schizophrenia; ASD, autism spectrum disorder. (d) Table indicating genomic coordinates of *PRRT2* duplications in four patients and their clinical features. Paroxysmal kinesigenic dyskinesia (PKD) is a rare paroxysmal movement disorder, often misdiagnosed as epilepsy, and characterized by recurrent, brief dyskinesia attacks triggered by sudden voluntary movement (MIM: 128200). (e) Genomic mapping of *PRRT2* duplications on human chromosome 16 using UCSC genome browser. The varying size of the duplications in each patient can be observed in the top part of the panel. All duplications encompass the entire *PRRT2* coding region marked by the consensus coding sequence (CCDS, green). All RefSeq transcripts proximal to the duplicated region are indicated (Blue). The level of conservation in 100 vertebrate species is also indicated and is highly correlated with coding regions (100 Vert. Cons.). The location of clinically relevant variants (ClinVar) and OMIM genes in the neighboring region is depicted in the bottom part of the panel.

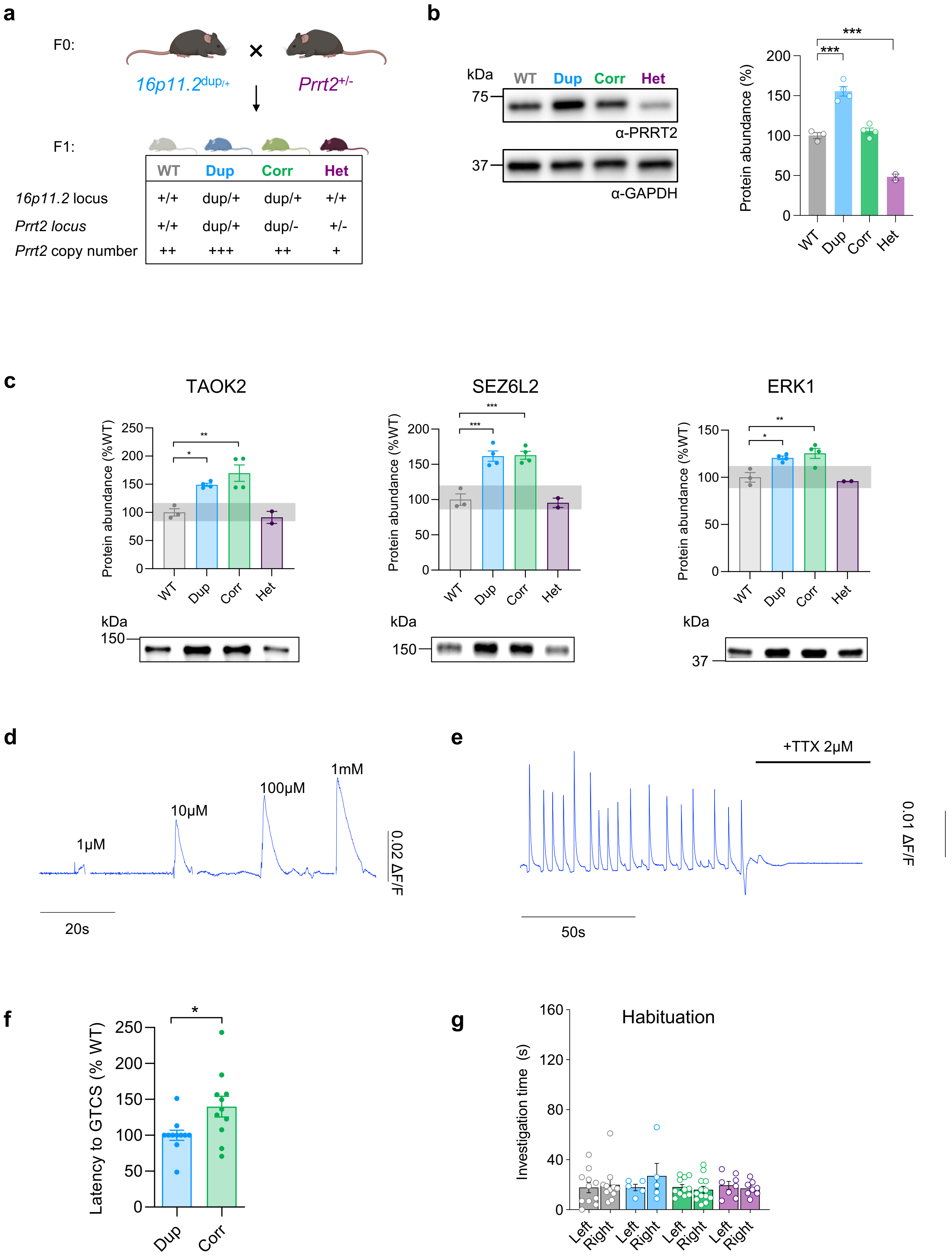

a
F0:
16p11.2dup/+
Prrt2+/-
F1:
WT Dup Corr Het
16p11.2 locus +/+ dup/+ dup/+ +/+
Prrt2 locus +/+ dup/+ dup/- +/-
Prrt2 copy number ++ +++ ++ +
b
kDa
WT Dup Corr Het
75
α-PRRT2
37
α-GAPDH
Protein abundance (%)
***
***
WT Dup Corr Het
c
TAOK2
SEZ6L2
ERK1
Protein abundance (%WT)
WT Dup Corr Het
kDa
150
37
d
1μM
10μM
100μM
1mM
0.02 ΔF/F
20s
e
+TTX 2μM
0.01 ΔF/F
50s
f
Latency to GTCS (% WT)
*
Dup Corr
g
Habituation
Investigation time (s)
Left Right Left Right Left Right Left Right

Supplementary Fig. 8. Additional data for experiments on 16p11.2$^{dup/+}$ mice corrected for *Prrt2* copy number. (a) Breeding scheme for generating *16p11.2*$^{dup/+}$ mice corrected for PRRT2 expression (*16p11.2*^{corr}). Mouse schematic created with BioRender.com. (b-c) Western blots of proteins encoded by genes in the *16p11.2* chromosomal region (WT n=3 mice, Dup n=4 mice, Corr n=4 mice, Het n=2 mice). Data are presented as mean ± s.e.m. (b) Western blots showing that PRRT2 expression levels in *16p11.2*^{corr} mice are similar to WT mice (WT vs Corr, p <0.8263; WT vs. Dup, p <0.0001; WT vs. Het, p = 0.0006, One-way ANOVA with Tukey posthoc test). (c) Corr mice have wild-type levels of PRRT2 expression but other proteins associated with the *16p11.2* region remain increased (TAOK2: WT vs. Dup, p = 0.0316; WT vs. Corr, p = 0.004; SEZ6L2: WT vs. Dup, p = 0.0007; WT vs. Corr, p = 0.0006; ERK1: WT vs. Dup, p = 0.0281; WT vs. Corr, p = 0.0087, One-way ANOVA with Tukey posthoc test). (d) Traces showing Syn-iGluSnFr sensitivity to increasing glutamate concentrations (infected into of cortical neurons). (e) Neuronal network activity measured by Syn-iGluSnFr is dependent on action potentials, as Na_V channel blocker TTX prevents fluorescence. (f) Corr mice have delayed seizure onset compare to Dup mice (Dup n=11 mice, Corr n=11 mice, p = 0.0227, two-sided t-test). (g) Mice in the social interaction task have no preference for either side during the habituation phase (WT, n=11 mice; Dup, n=5 mice; Corr, n=13 mice; Het, n=8 mice). Data are presented as mean ± s.e.m. Abbreviations: WT, *16p11.2*$^{+/+}$; Dup, *16p11.2*$^{dup/+}$; Corr, *16p11.2*^{corr}; Het, *Prrt2*$^{+/-}$. *p < 0.05, **p< 0.01, ***p< 0.001
