# Supplementary material for: Rescue of neuropsychiatric phenotypes in a mouse model of 16p11.2 duplication syndrome by genetic correction of an epilepsy network hub
Source: Nat Commun. 2023 Feb 17;14:825. doi: 10.1038/s41467-023-36087-x (PMC9938216; doi:10.1038/s41467-023-36087-x)
Supplement: Supplementary file 3 — Description of Additional Supplementary Files [file 41467_2023_36087_MOESM3_ESM.pdf]

Description of Additional Supplementary Files

Supplementary Data 1. Full ^{15}N membrane proteome dataset from $16p11.2^{dup/+}$ mouse model.

Supplementary Data 2. GO cluster analysis of dysregulated proteins in cortical membranes from 16p11.2 duplication mice.

Supplementary Data 3. SynGO analysis of dysregulated proteins in 16p11.2 duplication cortical membrane proteome

Supplementary Data 4. GO cluster analysis of primary epilepsy-associated network

Supplementary Data 5. PRRT2 interactome dataset (IAP-MS)

Supplementary Data 6. GO cluster analysis of PRRT2 interactome dataset

Supplementary Data 7. SynGO analysis of PRRT2 interactome dataset

Supplementary Movie 1. Two-photon calcium imaging of layer 2/3 somatosensory cortex from (Left) $16p11.2^{+/+}$ mice and (Right) $16p11.2^{dup/+}$ mice.

Supplementary Movie 2. Glutamate imaging of cortical networks in wild-type and $16p11.2^{dup/+}$ mice.
