# Supplementary material for: Rescue of neuropsychiatric phenotypes in a mouse model of 16p11.2 duplication syndrome by genetic correction of an epilepsy network hub
Source: Nat Commun. 2023 Feb 17;14:825. doi: 10.1038/s41467-023-36087-x (PMC9938216; doi:10.1038/s41467-023-36087-x)
Supplement: Supplementary file 7 — Reporting Summary [file 41467_2023_36087_MOESM7_ESM.pdf]

nature portfolio

Corresponding author(s): Peter Penzes
Last updated by author(s): Dec 12, 2022

Reporting Summary

Nature Portfolio wishes to improve the reproducibility of the work that we publish. This form provides structure for consistency and transparency in reporting. For further information on Nature Portfolio policies, see our Editorial Policies and the Editorial Policy Checklist.

Statistics

For all statistical analyses, confirm that the following items are present in the figure legend, table legend, main text, or Methods section.

n/a	Confirmed	
☐	☒	The exact sample size (*n*) for each experimental group/condition, given as a discrete number and unit of measurement
☐	☒	A statement on whether measurements were taken from distinct samples or whether the same sample was measured repeatedly
☐	☒	The statistical test(s) used AND whether they are one- or two-sided<br>*Only common tests should be described solely by name; describe more complex techniques in the Methods section.*
☒	☐	A description of all covariates tested
☐	☒	A description of any assumptions or corrections, such as tests of normality and adjustment for multiple comparisons
☐	☒	A full description of the statistical parameters including central tendency (e.g. means) or other basic estimates (e.g. regression coefficient) AND variation (e.g. standard deviation) or associated estimates of uncertainty (e.g. confidence intervals)
☐	☒	For null hypothesis testing, the test statistic (e.g. *F*, *t*, *r*) with confidence intervals, effect sizes, degrees of freedom and *P* value noted<br>*Give P values as exact values whenever suitable.*
☐	☒	For Bayesian analysis, information on the choice of priors and Markov chain Monte Carlo settings
☒	☐	For hierarchical and complex designs, identification of the appropriate level for tests and full reporting of outcomes
☐	☒	Estimates of effect sizes (e.g. Cohen's *d*, Pearson's *r*), indicating how they were calculated

Our web collection on statistics for biologists contains articles on many of the points above.

Software and code

Policy information about availability of computer code

Data collection	Mass spectrometry: EASY nLC 1000, Orbitrap Fusion with Xcalibur 4.4, IP2 6.5.5, RawConverter 1.0.0.0<br>Imaging: Nikon C2+ microscope or Nikon A1R MP with NIS-Elements v4.20<br>Western Blot: Image Lab v6.1.0<br>Behavior: LimeLight v2.3
Data analysis	Western blot: Image Lab v6.1.0<br>Image analysis: NIS-Elements v4.20, Image J (FIJI v2.0.0), MATLAB R2020a, update 6<br>Bioinfomatics: Cytoscape v3.6.0, DAVID v6.8, SynGO 1.1 (20210225)<br>Behavior: Ethovision v12<br>Statistics: GraphPad Prism v9.3.1, R studio 1.2.1335

For manuscripts utilizing custom algorithms or software that are central to the research but not yet described in published literature, software must be made available to editors and reviewers. We strongly encourage code deposition in a community repository (e.g. GitHub). See the Nature Portfolio guidelines for submitting code & software for further information.

Data

Policy information about availability of data

All manuscripts must include a data availability statement. This statement should provide the following information, where applicable:

- Accession codes, unique identifiers, or web links for publicly available datasets
- A description of any restrictions on data availability
- For clinical datasets or third party data, please ensure that the statement adheres to our policy

There are no restrictions on data availability in this manuscript. All data generated in this study are included in the article (and its supplementary information files). Mass spectrometry data has been deposited to MassIVE (MSV000090884) and ProteomeXchange (PXD038753).

Human research participants

Policy information about studies involving human research participants and Sex and Gender in Research.

Reporting on sex and gender	N/A
Population characteristics	N/A
Recruitment	N/A
Ethics oversight	N/A

Note that full information on the approval of the study protocol must also be provided in the manuscript.

Field-specific reporting

Please select the one below that is the best fit for your research. If you are not sure, read the appropriate sections before making your selection.

☒ Life sciences ☐ Behavioural & social sciences ☐ Ecological, evolutionary & environmental sciences

For a reference copy of the document with all sections, see nature.com/documents/nr-reporting-summary-flat.pdf

Life sciences study design

All studies must disclose on these points even when the disclosure is negative.

Sample size	Sample sizes were selected based on previously published work from our lab using similar techniques (PMID: 34921780, PMID: 34921780)
Data exclusions	only one data point was excluded (Fig. 5F). The outlier was removed based on a Grubb's test at alpha=0.01
Replication	All experimental findings were replicated at least 3 times. IP-WB experiments (Figure S6) were performed once as these were considered validations of the IP-MS experiment (Figure 4). All other data were from at least 4 mice (from at least 2 independent litters). All replicated data were pooled and assessed for statistical significance. Individual replications were not tested for statistical significance because of insufficient power.
Randomization	For imaging and behavioral analysis, individual plates (imaging) or cages (behaviour) were labelled at random number and processed in ascending order.
Blinding	All experimenters were blinded to genotype during the data collection and analysis phases.

Reporting for specific materials, systems and methods

We require information from authors about some types of materials, experimental systems and methods used in many studies. Here, indicate whether each material, system or method listed is relevant to your study. If you are not sure if a list item applies to your research, read the appropriate section before selecting a response.

Materials & experimental systems

n/a	Involved in the study
☐	☒ Antibodies
☐	☒ Eukaryotic cell lines
☒	☐ Palaeontology and archaeology
☐	☒ Animals and other organisms
☒	☐ Clinical data
☒	☐ Dual use research of concern

Methods

n/a	Involved in the study
☒	☐ ChIP-seq
☒	☐ Flow cytometry
☒	☐ MRI-based neuroimaging

Antibodies

Antibodies used: SEZ6L2 (Abcam, ab197058, 1:500), PRRT2 (Sigma, HPA014447, 1:2000), ERK1 (Santa Cruz, sc94, 1:5000), and TAOK2 (Santa Cruz, sc-47447, 1:500), FLAG (Sigma ,F1804, 1:1000), anti-T7 (Millipore, AB3790, 1:1000), anti-Myc (Santa Cruz, sc-789, 1:1000)

Validation: PRRT2 and SEZ6L2 were validated in house by overexpressing cDNA constructs containing target genes in HEK293 cells, followed by western blotting of cell lysates. FLAG, T7 and Myc epitope tag antibodies were also validated in house using overexpression constructs and western blotting. ERK1 is a highly cited antibody and was used previously (PMID: 27402753 and https://www.scbt.com/p/erk-1-antibody-k-23). TAOK2 was used in a previous publication (PMID: 22683681).

Eukaryotic cell lines

Policy information about cell lines and Sex and Gender in Research

Cell line source(s): HEK293-T cells (ATCC #CRL-11268)

Authentication: None of the lines used were authenticated.

Mycoplasma contamination: Cell lines were not routinely tested for Mycoplasma contamination but were provided Mycoplasma free from the vendor and frozen in aliquots.

Commonly misidentified lines (See ICLAC register): No commonly misidentified cell lines were used in this study

Animals and other research organisms

Policy information about studies involving animals; ARRIVE guidelines recommended for reporting animal research, and Sex and Gender in Research

Laboratory animals: Strain: Mus musculus C57BL/6
Genetically modified mice: 16p11.2 dup (Jackson Lab, Stock No. 016915), PRRT2+/- (LeDoux lab)
Sex: Male and female mice were used for all experiments except the initial MS experiment in Figure 1, where only males were used.
Age: For behavioral experiments, all mice were between 6-10 weeks of age
Environment: 14-h on/10-h off light/dark cycle with temperature 70-74F and humidity 30-70%

Wild animals: This study did not involve wild animals.

Reporting on sex: Sex was not considered in the study design. However, we did not observe any obvious sex-specific differences in behavior post-hoc. Please see figure S4 for effect of sex on seizure susceptibility (males and females respond similarly).

Field-collected samples: This study did not involve samples collected from the field

Ethics oversight: All protocols for animal experiments were approved by the Institutional Animal Care and Use Committee (IACUC) at Northwestern University.

Note that full information on the approval of the study protocol must also be provided in the manuscript.
